# Basal cell transplantation supports multilineage differentiation and restores CFTR function in ex vivo ferret airways

**DOI:** 10.1172/jci.insight.206375

**Published:** 2026-08-18

**Authors:** Kadambari Vijaykumar, Liang Ma, Kevin Chen, Liping Tang, Nikoleta Pavelkova, Elex Harris, Kajal Jadhav, Qian Li, Mohamed Hanafy, Hinnerk Schulz-Hildebrandt, Guillermo J. Tearney, Finn Hawkins, Darrell N. Kotton, Steven M. Rowe

**Affiliations:** 1 Department of Medicine and Gregory Fleming James Cystic Fibrosis Research Center, University of Alabama at Birmingham, Birmingham, Alabama, USA.; 2 Center for Regenerative Medicine, Boston Medical Center, Boston, Massachusetts, USA.; 3 Wellman Center for Photomedicine, Massachusetts General Hospital, Boston, Massachusetts, USA.

**Keywords:** Cell biology, Pulmonology, Stem cell transplantation

## Abstract

This study uses ferret cells and tissues to find what stem cells need to successfully repair airway damage for further application in cystic fibrosis.

**To the Editor:** Cell-based therapies offer a mutation-agnostic approach to restoring airway function in cystic fibrosis (CF), overcoming limitations of genotype-specific modulators (1). Basal cells are central to airway repair (2) owing to their capacity for self-renewal and multilineage differentiation (3). While basal cells have durably reconstituted rodent airway epithelium (4), functional disease rescue following cell engraftment has not been shown. To enable in vivo translation, proof-of-concept testing in models that recapitulate CF pathophysiology is essential. Here, we utilized ferret bronchial epithelial cells (FBECs) in what we believe to be novel in vitro and ex vivo systems to define technical and physiological benchmarks for cellular reconstitution.

## In vitro injury and rescue

Primary FBECs from WT and CF G551D ferrets were harvested by bronchoscopy, expanded, and differentiated at air-liquid interface (ALI). WT FBECs were treated with polidocanol (PDOC) to generate a permissive cell niche. Epithelial integrity was monitored via transepithelial electrical resistance (TEER), trypan blue viability assays, and microscopy. The highest concentrations of PDOC (0.125% and 0.25%) caused the highest degree of cell death. Conversely, 0.03% and 0.05% PDOC maintained high viability (77%–81%) but insufficient niche generation for cell implantation (TEER drop <50% at 60 minutes). 0.1% PDOC yielded approximately 59% live cells, with 95% TEER drop balancing niche generation with cell viability, compared to 0.04% PDOC required to restore barrier integrity in human cells (5).

Injury kinetics of 0.1% PDOC were similar between WT and CF G551D ALI cultures (*n* = 9 each) with rapid decline in TEER over 30 minutes. In WT cells, baseline TEER (2,200–3,000 Ω × cm^2^) decreased (to <400 Ω × cm^2^) within 20 minutes of PDOC exposure, which returned to baseline by day 12. CF G551D cells had lower baseline TEER (~1,400 Ω × cm^2^) but showed similar TEER decline and recovery (Figure 1A). These results confirm that 0.1% PDOC generates a permissive niche without hindering regeneration.

To evaluate functional rescue, early passage WT FBECs were GFP labeled by lentiviral transduction and then delivered (1.5 × 10^5^ cells in 100 μL PneumaCult-EX Plus media) onto CF G551D-differentiated cultures 30 minutes after 0.1% PDOC. At 4 weeks, fluorescence microscopy (Figure 1D) and micro-optical coherence imaging confirmed engraftment, comprising about 77% of epithelium. Short-circuit current (Isc) analysis by Ussing at 4 weeks after implantation confirmed CF transmembrane conductance regulator (CFTR) rescue in CF cultures with WT transplantation, restoring forskolin responsiveness (mean ΔIsc ± SD, after forskolin 121.5 ± 10.1 μA/cm^2^, after CFTR_Inh_-172/GlyH101 –84.8 ± 0.9 μA/cm^2^) compared with PDOC-only (7.9 ± 2.6 μA/cm^2^ after forskolin, *P* < 0.004; 8.5 ± 1.8 μA/cm^2^ after CFTR_Inh_-172/GlyH-101, *P* < 0.0004) and WT PDOC controls (82.1 ± 7.4 μA/cm^2^ after forskolin, 67.9 ± 9.5 after CFTR_Inh_-172/GlyH-101; *n* = 3/condition) (Figure 1, B and C). WT to CF transplant FBECs showed significant improvement in mucociliary transport (MCT; mean ± SD, 1.2 ± 0.1 mm/min), comparable to WT to WT transplant controls (1.1 ± 0.5 mm/min) (Figure 1E). Collectively, these data indicate successful rescue of CFTR function and mucus transport in CF G551D FBECs through implantation of WT cells.

## Ex vivo tracheal engraftment

Excised WT ferret tracheae were cultured on a surgifoam/gelatin matrix in a Petri dish with antimicrobial-enriched PneumaCult-EX Plus media. Following niche generation by mechanical brushing (~15 strokes across tracheal surface using cytology brush), GFP^+^ WT FBECs (20 × 10^6^ cells in 200 μL DMEM administered by pipette) were seeded (Figure 1F). At 3 weeks, histology (Figure 1G) and immunofluorescence detected successful GFP^+^ cell engraftment (*N* = 4 replicates), with approximately 30% of surface epithelial cells expressing GFP. IF analysis (Figure 1H) of the engrafted tissue demonstrated differentiation of GFP^+^ cells into ciliated (acetylated tubulin), secretory (MUC5B), and basal (KRT5) cell types. Ussing chamber analysis (Figure 1, I and J) demonstrated CFTR-dependent activation and inhibition in engrafted trachea at 3 weeks ALI (mean ΔIsc ± SD, 361 ± 57.5 μA/cm^2^ after forskolin, –788.5 ± 137.1 μA/cm^2^ after CFTR_Inh_-172/GlyH101) in comparison with injury-only (138.5 ± 116.7 μA/cm^2^ after forskolin, –205 ± 237.6 μA/cm^2^ CFTR_Inh_-172/GlyH101) and untreated controls (1,060 ± 315.4 μA/cm^2^ after forskolin, –979 ± 496.4 μA/cm^2^ CFTR_Inh_-172/GlyH101; *n* = 4/condition). Ciliary function was demonstrated (Figure 1K). These data demonstrate that transplanted cells can achieve multilineage differentiation and sustain CFTR-dependent ion transport and mucociliary function in a tissue culture model.

Together, these findings provide proof of concept that transplanted FBECs can engraft, undergo multilineage differentiation, and restore CFTR-dependent mucociliary function in vitro and ex vivo. These platforms establish critical benchmarks for injury, cell delivery, and functional assessment, paving way for in vivo translation. Importantly, use of different injury patterns — chemical in ALI cultures and mechanical in ex vivo tracheae — underscores the need to tailor niche generation. Ultimately, this work supports cell transplantation as a scalable, mutation-agnostic strategy for durable CF airway repair, supporting future studies to elucidate factors required to successfully restore normal physiology.

For detailed methods, information regarding sex as a biological variable, statistics, study approval, data availability, author contributions, and acknowledgments, see the supplemental materials (supplemental material available online with this article; https://doi.org/10.1172/jci.insight.206375DS1).

## Funding support

This work is the result of NIH funding, in whole or in part, and is subject to the NIH Public Access Policy. Through acceptance of this federal funding, the NIH has been given a right to make the work publicly available in PubMed Central.

- NIH (2P30DK072482 to University of Alabama at Birmingham).
- Cystic Fibrosis Foundation (ROWE24XX1, VIJAYK25XX0).

## Conflict of interest

The authors have declared that no conflict of interest exists.

## Supplementary Material

Supplemental data

Supporting data values

## Figures and Tables

**Figure 1:**
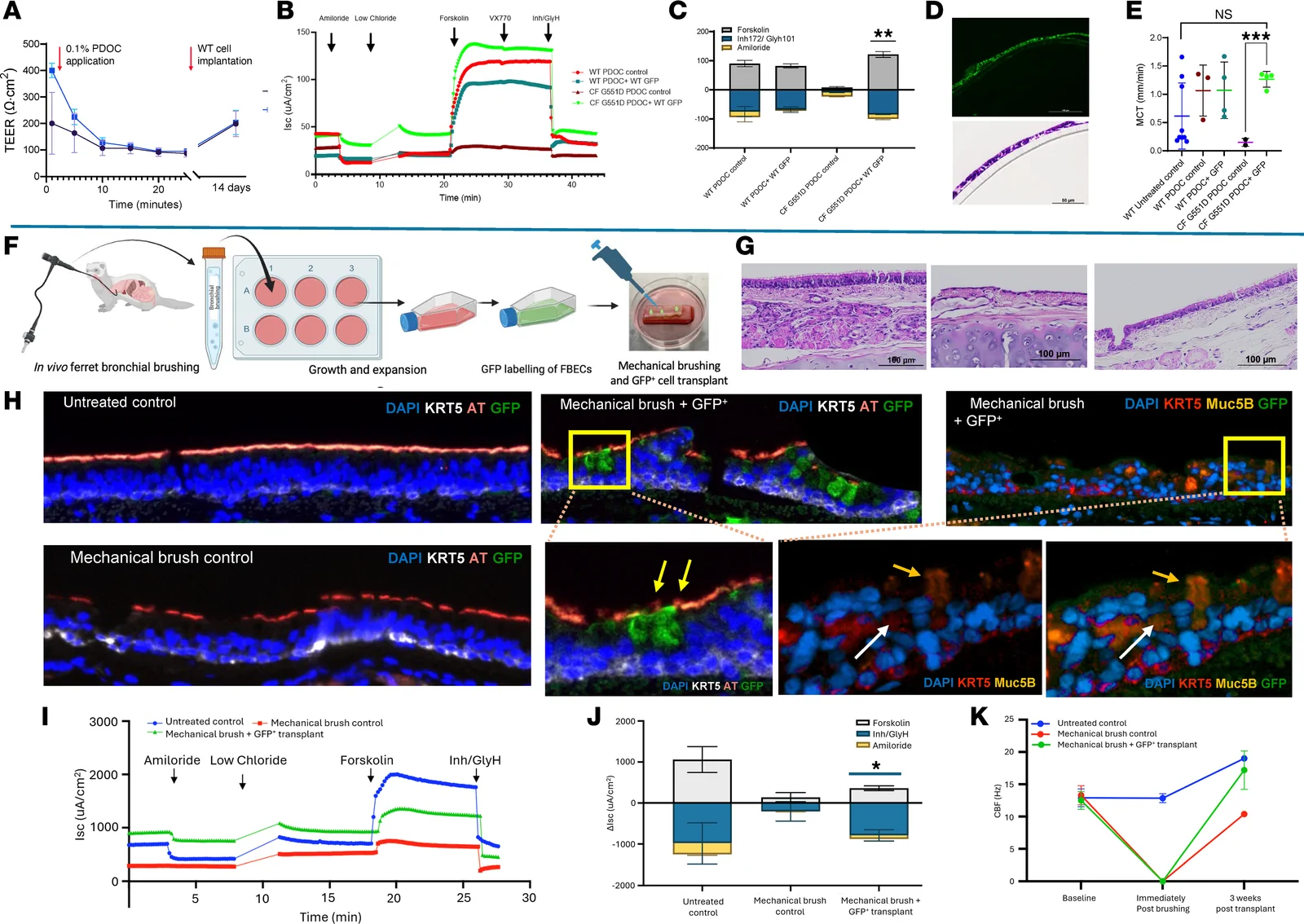
Functional reconstitution of ferret airway epithelium following basal cell transplantation. (**A**–**E**) In vitro assessment. (**A**) Transepithelial electrical resistance (TEER) with decline of barrier integrity following 0.1% polidocanol (PDOC) exposure and recovery following implantation. (**B**) Using chamber traces of WT and CFG551D FBECs 4 weeks after injury and GFP^+^ cell implantation. (**C**) Change in short-circuit current (ΔIsc) after forskolin with CFTR functional rescue in transplanted CF cultures (*P* = 0.0024). (**D**) Fluorescent microscopy confirms incorporation of GFP^+^ donor cells into epithelium. Scale bars: 50 μm. (**E**) Micro-optical coherence tomography (μOCT) shows improvement in mucociliary transport in transplanted CF cultures (*P* = 0.0005). (**F**–**K**) Ex vivo tracheal engraftment. (**F**) FBEC harvest, expansion, and transplant protocol. (**G**) H&E staining showing intact ciliation in untreated control (left) compared with ciliary loss and basal cell exposure with injury (middle) and evidence of differentiation after transplant (right). Scale bars: 100 μm. (**H**) Immunofluorescence microscopy showing GFP^+^ cell engraftment (middle and right, top row) 3 weeks after transplant compared with controls (left, top and bottom row). GFP^+^ FBECs differentiate into ciliated (second image, bottom row, yellow arrows, GFP^+^AT^+^), secretory (third image, bottom row, orange arrow, MUC5B only; last image, bottom row, costained with GFP and MUC5B), and basal cells (last image, bottom row, white arrow, KRT5^+^GFP^+^). (**I**) Isc responses under chloride secretory gradient conditions and (**J**) summary data (ΔIsc, *n* = 4) with recovery of CFTR function in engrafted tissue (*P* = 0.038). (**K**) μOCT showing improved ciliary beat frequency in transplanted tissue (*P* = 0.0049). Data presented as mean ± SD. Statistical significance determined using 1-way ANOVA: *P < 0.05; **P ≤ 0.01; ***P ≤ 0.001.

## Version 1 08/18/2026

In-Press Preview

## Version 2 10/08/2026

Electronic publication
