# Supplementary material for: Basal cell transplantation supports multilineage differentiation and restores CFTR function in ex vivo ferret airways
Source: JCI Insight. 2026 Aug 18;11(19):e206375. doi: 10.1172/jci.insight.206375 (PMC13726407; doi:10.1172/jci.insight.206375)
Supplement: Supplementary file 1 [file jciinsight-11-206375-s002.pdf]

SUPPLEMENTAL MATERIAL

Methods

In vivo ferret bronchoscopy

Bronchoscopy was performed per the institutional IACUC approvals (#22877). Following moderate sedation, a slim bronchoscope was introduced through oropharynx, passed through the vocal cords, and airway inspected. Using a cytology brush inserted through the working channel of the bronchoscope, bronchial brushings were obtained from each of the main lobar bronchi bilaterally. The ferret bronchial epithelial cells (FBECs) are then expanded in culture to air-liquid interface (ALI).

Lentivirus GFP labelling

Primary FBECs obtained through bronchial brushing were maintained in PneumaCult-EX+ expansion medium (StemCell Technologies) supplemented with Primocin, Y-27632 (ROCK inhibitor), hydrocortisone, DMH-1, and A83-01 as previously published.[1] To prepare for transduction, FBECs were cultured in 2D on plastic in 6-well plates until they reached approximately 80% confluence. Cells were washed with PBS and dissociated into a single-cell suspension using 1 mL of ice-cold Accutase (Millipore Sigma) for 15–20 minutes at 37°C. Following dissociation, cell numbers were determined via hemacytometer to calculate the precise viral volume required for the target multiplicity of infection (MOI). Transduction was performed using a pHAGE-EF1aL-GFP-W lentivirus at an MOI of 20 as previously described.[2] Viral particles were delivered in a 500uL volume of supplemented PneumaCult-EX+ medium containing 5 ug/mL polybrene. After an overnight incubation at 37ºC, the viral medium was aspirated, and the cells were washed with 2mL of PBS to remove residual debris and viral particles. Cells were further expanded in fresh supplemented PneumaCult-EX+ medium until confluent and subsequently passaged. Transduction efficiency was validated by quantifying GFP expression via fluorescence-activated cell sorting (FACS) during passage against a polybrene-only treatment (no virus) control well to set baseline gating parameters for FACS analysis.

Ex vivo tracheal culture

This method was adopted and developed based on previous rat tracheal culture protocols.[3] Briefly, trachea was exposed from the larynx to the carina, excised, and cleaned of excess tissue. It was immediately incubated on ice in 5mL Pneumacult media (with PenStrep and AmpB) for one hour to equilibrate and reduce microbial load. After incubation, the trachea was transferred to a sterile hood and oriented by its dorsal side. It was then cut longitudinally from the distal to the proximal end. The tissue was placed ventral-side down onto Surgifoam™ sponges atop silicone strips within a culture cassette. The specimen was secured by pinning 27g needles through the outer walls into the silicone strip, ensuring the tissue was flattened without tension to maximize surface contact with the sponge (Fig 1). Finally, the trachea was rinsed three times with cold DPBS, and the cassette was filled with media until it reached the sponge level to ensure hydration.

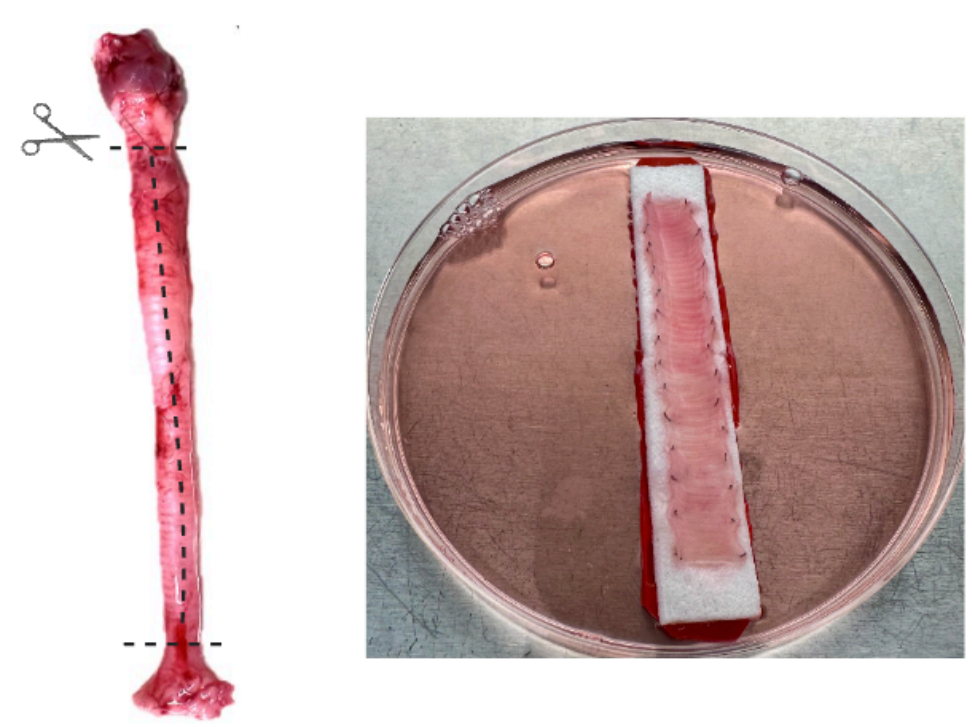

Figure 1: Excision and fixation of ferret trachea for tissue culture

Short-circuit current measurements on cultured primary ferret bronchial epithelial cells and ex vivo ferret trachea

Short-circuit current (Isc) was measured under voltage clamp conditions using MC8 clamps and P2300 Ussing chambers (Physiologic Instruments, San Diego, CA). Using *P2302T* mounting chambers (aperture size 6.5mm and 0.33cm^2 exposed surface area) that are specifically designed for transwell inserts. ALI monolayers were initially bathed on both sides with identical Ringer's solutions containing (in mM) 120 NaCl, 25 $NaHCO_3$, 3.33

KH_2PO_4, 0.83 K_2HPO_4, 1.2 $CaCl_2$, 1.2 $MgCl_2$, and 10 D-glucose (pH 7.4). Bath solutions were vigorously stirred and gassed with 95%O2:5% CO_2. A one-second three-mV pulse was imposed every 10 seconds to monitor resistance calculated using Ohm's law. Where indicated, the mucosal bathing solution was changed to a low Cl− solution containing 1.2 NaCl and 120 Na+ gluconate, and all other components as above. This was followed by the CFTR agonist forskolin as indicated with minimum five-min observation at each concentration. GlyH101 (20 µM final concentration) was added to the mucosal bathing solution at the end of experiments to block CFTR-dependent Isc. All chambers were maintained at 37°C.
Tracheal short circuit current studies to assess ion transport across the epithelium were conducted on freshly harvested trachea from euthanized ferrets. Isc measurements in ferret airway explants were conducted following dissection of the mucosal layer and were performed as in ALI monolayers, except for use of *P2306* tissue mounts (aperture size 2.8 x 1.5mm and 0.04cm^2 exposed surface area). Amiloride (100 µM) was added to block residual Na+ current and was followed by a low Cl− solution containing 1.2 NaCl and *120* Na+ gluconate. The CFTR agonist forskolin and antagonist GlyH101 were added to the mucosal bathing solution, where indicated, to respectively activate and thereafter block CFTR-dependent Isc.

Micro-optical coherence tomography
The ThorImage micro-optical coherence (µOCT) system supports end-to-end imaging workflows, including acquisition, processing, visualization, and data management. High-speed image capture is enabled by a dedicated frame grabber interface (Matrox-based acquisition hardware/drivers), which efficiently streams imaging data to the computer, and it leverages a high-performance CPU/GPU workstation for responsive processing and real-time visualization. The imaging module uses an industrial-grade, board-level CMOS camera with 5 MP resolution (2592 × 1944) and a 1/2.5″ sensor (2.2 µm pixel size), providing high resolution spatial detail for imaging and analysis. The system supports both 2D and 3D scanning, with acquisition times on the order of tens of milliseconds (approximately 0.012–0.025 s, depending on the selected field of view). This interface provides real-time visualization during acquisition, enabling rapid assessment of signal quality and alignment to minimize repeat scans. The main parameters assessed were airway surface liquid (ASL), periciliary liquid (PCL), mucociliary transport (MCT), and ciliary beat frequency (CBF). ASL and PCL thickness measurements were obtained from OCT image sequences in Image J using geometric measurements of the visible layer boundaries as in several publications. MCT was estimated from resliced OCT kymographs by tracking mucus-associated particle displacement across sequential frames and converting the measured motion into transport velocity. CBF was determined using pixel-wise temporal fast Fourier transform (FFT) analysis of sequential images, with the dominant spectral peak used to estimate beat frequency. The MATLAB code for this analysis was developed and maintained by the authors at UAB and MGH and validated in several publications[4-8].

Histology and immunofluorescent staining
Ferret tracheae were fixed with 4% PFA for 2 hours at room temperature and washed 3 times for 10min with PBS. Tracheae were embedded in paraffin and sectioned to 5µm sections. Formalin fixed paraffin embedded sections of ferret trachea on slides were melted in dry oven at 60°C for 1h, deparaffinized in 2 changes of xylene, rehydrated in reverse ethanol gradient, washed in deionized water and PBS. Antigen retrieval was performed in 0.01M Tris-1mM EDTA buffer (pH 9) at 65°C for 30min, followed by rinsing in deionized water. Tissue sections were permeabilized in PBS + 0.3% Triton X-100 for 15min at room temperature, followed by blocking with 5% normal donkey serum for 1h at room temperature, they were then incubated in primary antibodies overnight at 4°C and in secondary antibodies 1h at room temperature. Slides were mounted with VECTASHIELD Vibrance Antifade mounting medium with DAPI and imaged with Lionheart FX automated microscope. The following primary and secondary antibodies were used: anti-GFP (Cat# 1010, Aves Labs, 1:500), anti-AT (Cat# T7451, Sigma-Aldrich, 1:15000), anti-KRT5 (Cat# ab64081, Abcam, 1:100), anti-Muc5B (Cat# HPA008246, Sigma-Aldrich, 1:400), anti-chicken IgY AF488 (Cat# A78948, Thermo Fisher, 1:1000), anti-mouse IgG AF594 (Cat# A21203, Thermo Fisher, 1:1000), anti-rabbit IgG AF594 (Cat# A21207, Thermo Fisher, 1:1000), anti-rabbit IgG AF647 (Cat# A31573, Thermo Fisher, 1:1000). All images were obtained from Lionheart FX (Biotek) in the UAB Cystic Fibrosis Research Center.

Study approval
The in vivo procedures conducted in this study are approved under the University of Alabama at Birmingham's Institutional Animal Care and Use Committee (IACUC #22877).

Statistics
Continuous variables were summarized as mean ± standard deviation or percentage where appropriate. Sample sizes ranged from n = 3 to n = 9 replicates per experimental group across in vitro ALI cultures and ex vivo tracheal tissues. Transepithelial electrical resistance was monitored longitudinally to assess epithelial integrity and recovery kinetics over time. Comparisons of short-circuit current and micro-optical coherence tomography metrics between treatment groups (e.g., wild-type control, injury control, and transplanted groups) were evaluated for using GraphPad Prism version 9.1.0 for MacOS (GraphPad Software, San Diego, CA) Unpaired t-tests and one-way ANOVA were used to ascertain statistical significance. Significance levels were reported using standard P-value thresholds *P<0.05; **P≤ 0.01; ***P≤ 0.001.

Sex-as-a-biological-variable
Our study examined samples from both male and female animals, and similar findings are reported for both sexes.

Data availability
The raw data used and/or analyzed during the current study are in the supporting data file.

Author contributions
KV, LT, FH, DNK and SMR conceived the study design; LM and KC performed lentiviral optimization; KV, LT, NP, EH, KJ, QL, MH, and HSH participated in experimental conduct and acquisition of data; KV, LT, NP, GJT, FH, DNK and SMR performed data analysis; KV and SMR prepared the manuscript.
All authors provided necessary revisions and approved a final draft prior to submission.

REFERENCES

1. Hawkins FJ, Suzuki S, Beermann ML, et al. Derivation of Airway Basal Stem Cells from Human Pluripotent Stem Cells. Cell Stem Cell 2021;28(1):79-95.e8. DOI: 10.1016/j.stem.2020.09.017.
2. Ma L, Thapa BR, Le Suer JA, et al. Life-long functional regeneration of in vivo airway epithelium by the engraftment of airway basal stem cells. Nat Protoc 2025;20(3):810-842. (In eng). DOI: 10.1038/s41596-024-01067-y.
3. Harris E, Easter M, Ren J, Krick S, Barnes J, Rowe SM. An ex vivo rat trachea model reveals abnormal airway physiology and a gland secretion defect in cystic fibrosis. Plos one 2023;18(10):e0293367.
4. Liu L, Chu KK, Houser GH, et al. Method for quantitative study of airway functional microanatomy using micro-optical coherence tomography. PLoS One 2013;8(1):e54473. DOI: 10.1371/journal.pone.0054473.
5. Lin VY, Kaza N, Birket SE, et al. Excess mucus viscosity and airway dehydration impact COPD airway clearance. Eur Respir J 2020;55(1). DOI: 10.1183/13993003.00419-2019.
6. Chu KK, Unglert C, Ford TN, et al. In vivo imaging of airway cilia and mucus clearance with micro-optical coherence tomography. Biomed Opt Express 2016;7(7):2494-505. DOI: 10.1364/BOE.7.002494.
7. Fernandez-Petty CM, Hughes GW, Bowers HL, et al. A glycopolymer improves vascoelasticity and mucociliary transport of abnormal cystic fibrosis mucus. JCI Insight 2019;4(8). DOI: 10.1172/jci.insight.125954.
8. Li Q, Vijaykumar K, Phillips SE, et al. Mucociliary transport deficiency and disease progression in Syrian hamsters with SARS-CoV-2 infection. JCI Insight 2023;8(1) (In eng). DOI: 10.1172/jci.insight.163962.
